# Severe COVID-19 in Alzheimer’s disease: APOE4’s fault again?

**DOI:** 10.1186/s13195-021-00858-9

**Published:** 2021-06-12

**Authors:** Nian Xiong, Martin R. Schiller, Jingwen Li, Xiaowu Chen, Zhicheng Lin

**Affiliations:** 1 Department of Neurology, Union Hospital, Tongji Medical College, Huazhong University of Science and Technology, Wuhan, 430022 Hubei China; 2 Nevada Institute of Personalized Medicine and School of Life Sciences, University of Nevada Las Vegas, Las Vegas, NV 89154 USA; 3 Department of Neurology, Shenzhen University General Hospital, Shenzhen, 518000 Guangdong China; 4 Laboratory for Psychiatric Neurogenomics, McLean Hospital, Harvard Medical School, Belmont, MA 02478 USA

**Keywords:** APOE4, Biomarker, Coronavirus, Comorbidity, Peripheral mechanisms, COVID-19

## Abstract

Challenges have been recognized in healthcare of patients with Alzheimer’s disease (AD) in the COVID-19 pandemic, given a high infection and mortality rate of COVID-19 in these patients. This situation urges the identification of underlying risks and preferably biomarkers for evidence-based, more effective healthcare. Towards this goal, current literature review and network analysis synthesize available information on the AD-related gene *APOE* into four lines of mechanistic evidence. At a cellular level, the risk isoform APOE4 confers high infectivity by the underlying coronavirus SARS-CoV-2; at a genetic level, *APOE4* is associated with severe COVID-19; at a pathway level, networking connects APOE with COVID-19 risk factors such as ACE2, TMPRSS2, NRP1, and LZTFL1; at a behavioral level, APOE4-associated dementia may increase the exposure to coronavirus infection which causes COVID-19. Thus, APOE4 could exert multiple actions for high infection and mortality rates of the patients, or generally, with COVID-19.

## Background

In the midst of the COVID-19 pandemic, patients with Alzheimer’s disease (AD), once infected by the underlying coronavirus SARS-CoV-2, are 5 times likely to die of this infectious disease [1]. In the absence of effective treatment, a mechanistic understanding of how the patients with AD become a vulnerable target of COVID-19 may guide evidence-based healthcare management and targeted therapeutics development. In the earlier literature, it was postulated that *APOE4* (italic for gene), a genetic risk factor for AD [2], would be a biomarker for severe COVID-19 [3]; others considered psychological and behavioral contributions [4]. This review aims to capitalize on the evolving literature and database resources and seek a fundamental or molecular understanding of the high vulnerability in patients with AD.

## Genetic evidence for APOE4 involvement in the vulnerability for COVID-19

There is evidence for the genetic contribution to comorbidity of other brain disorders with COVID-19 [5] so that genetics may explain the high vulnerability of patients with AD as well. *APOE4* is the most established genetic risk factor for late-onset AD. An in vitro study has suggested that cells expressing APOE4 are more vulnerable to SARS-CoV-2 infection than those expressing the nonpathogenic isoform APOE3 [6]. Via induced pluripotent stem cells (iPSC)-based in vitro technologies, cells with APOE4 allowed more significant SARS-CoV-2 infection of artificially differentiated either neurons or astrocytes than those with APOE3. Furthermore, APOE4 astrocytes infected with SARS-CoV-2 presented a more severe cytopathogenic effect than APOE3 astrocytes, which could facilitate the progression and severity of COVID-19. This study indeed provided the first insight to a possible APOE-mediated mechanism for COVID-19 severity. It remained unknown how this genetic vulnerability was achieved and more importantly what this finding meant for a high infection rate in APOE4 carriers and increased COVID-19 mortality in the comorbid patients.

The difference between APOE4 and APOE2/3 is caused by the single nucleotide polymorphism (SNP) rs429358. This SNP carries two alleles, T and C, where T encodes a cystine (APOE3) and C encodes an arginine (APOE4) at residue position 130. In APOE4 carriers, reduced expression levels of APOE in both brain and peripheral systems suggested that this variant causes an increased risk not only for AD [7, 8], but also for systemic susceptibility to coronavirus infection. APOE4 has a worldwide average frequency of 15%, according to the 1000 Genomes Project [9], meaning that approximately 2% of the worldwide population are homozygotes, equal to the current 2% of the world population that have been diagnosed with COVID-19. In fact, APOE is abundantly expressed in cells of various peripheral systems including macrophages and epithelial cells of the lung [10, 11] which confers the most severe impact on coronavirus pathology. As an explanation, patients with APOE4-associated AD may carry higher vulnerability in their peripheral organs such as the lung than those carrying APOE3, possibly via enhancing the receptors’ activities and facilitating the coronavirus entry.

## Pathway support for a role of APOE4 in the severity of COVID-19

**Fig. 1:**
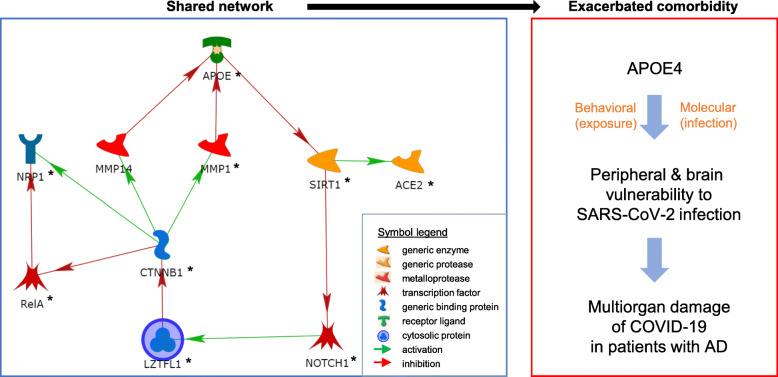
Possible mechanisms of APOE4-mediated AD and COVID-19 comorbidity. *Left panel*: A ten-member network shared by both AD (all members) and COVID-19 (*), generated by using MetaCore. In the case of APOE4, reduced/altered APOE has three potential actions in this network alone: (1) disinhibition of ACE2, (2) transcriptional reduction of the protective LZTFL1, and (3) more indirect disinhibition of NRP1 via LZTF1, in exacerbation of COVID-19. Asterisks are for genetic association with severe COVID-19: APOE: rs429358 (APOE2/3 vs 4) p = 0.0026, OR = 1.31; ACE2: chr23:15564667 p = 0.0056, OR = 1.12; CTNNB1: chr3:41204313 p = 0.016, OR = 0.74; LZTFL1: chr3:45834967 p = 1.15 × 10^−10^, OR = 0.56; NOTCH1: chr9:136510909 p = 0.0092, OR = 0.87; MMP1: rs11621460 p = 0.010, OR = 0.84; NRP1: chr10:33292184 p = 0.00072, OR = 1.47; RelA: rs1049728 p = 0.0063, OR = 0.64 (II); SIRT1: rs12783242 p = 0.0019, OR = 0.78; where chromosome positions are based on HG38 in the absence of rs numbers; OR, odds ratio; II, adjusted with gender and age; all association signals are provided by the GWAS meta-analysis by Ellinghaus et al. Not shown here is the additional APOE-LRP1-PARP1-TMPRSS2 pathway (see text): LRP1: rs4759044 p = 0.023, OR = 0.89; PARP1: chr1:226405149 p = 0.0065, OR = 0.51 (II). *Right panel*: APOE4-based vulnerability for patients with COVID-19 and AD at double risk: attenuated protective behavior for exposure risk and APOE4-associated infection risk

To explore this possibility, we have used a pathway analysis approach in MetaCore as previously described [12]. Results from this pathway analysis indeed support that possibility by identifying a plausible ten-member network where all ten members have been implicated in AD: APOE [13], ACE2 [14], CTNNB1 [15], NOTCH1 [16, 17], LZTFL1 [18], MMP1 [19], NRP1 [20], RELA [21], SIRT1 [22], and MMP14 [19] (*left panel* in Fig. 1). Among them, ACE2 and NRP1 are utilized by SARS-CoV-2 in order to enter cells and cause COVID-19 [23, 24]. ACE2 is almost undetectable and NRP1 has low expression levels in the brain. For a better understanding of the epidemiological finding, these functional genetic and pathway findings may encourage and re-direct our attention from the brain with AD to comorbid patients’ peripheral systems, where both receptors are well expressed and the coronavirus has an easy access, does extensive damages during the progression, and causes multiorgan damage-triggered mortality of COVID-19. Of note, it has been experimentally shown that the coronavirus (SARS-CoV) enters cells by binding to ACE2 while ACE2 may recycle subsequently back to cell surface after unloading of the virus [25]. Specifically, ACE2-related endocytosis, besides direct membrane fusion, has been proposed as an entry mechanism [26], consistent with the recent identification of the endosomal protein TMEM106B as another risk factor for the coronavirus infection [27].

Further pathway analyses found that SIRT1, the direct target of APOE, might regulate *TMEM106* through binding to several AD-related transcription factors (e.g., FOXP3 [28], STAT1 [29], BMAL1 [30, 31], SIRT6 [32], and E2F1 [33]) but the specifics on these regulations and on how TMEM106B regulates coronavirus’ intracellular activity remain to be uncovered. Interestingly, FOXP3 also bound to *TMPRSS2* which encoded another cell surface risk factor for SARS-CoV-2 infection [23]. Also, reduced APOE could reduce LRP1 inhibition of the TMPRSS2 activator PARP1, which in turn promotes the coronavirus infection as well (detailed pathway not shown) [34, 35]. Therefore, APOE might regulate the infectivity of SARS-CoV-2 in multiple ways.

Among the ten members, LZTFL1 represents the most significant genetic risk factor for severe COVID-19 as per findings from two genome-wide association studies (GWAS) of severe COVID-19 [18, 36]. Specifically, a minor allele of a SNP (G/GA, without a “rs” number yet) at chr3:45834967 in *LZTFL1* was protective against progression to severe forms of COVID-19. Together, APOE4 may have tetrad action: it enhances ACE2 activity by disinhibiting SIRT1 (Sirtuin 1, a generic enzyme), activates TMPRSS2 by the LRP1-PARP1 pathway, decreases the LZTFL1 expression by inhibiting NOTCH1, and activates NRP1 via LZTFL1 indirectly, satisfying the protective roles of both APOE and LZTFL1. That is, reduced APOE levels, which have demonstrated to be associated with the increased risk of AD, may disinhibit ACE2, TMPRSS2, and NRP1 and consistently increase the vulnerability to the coronavirus infection in the patients with AD. In fact, indirect activation of APOE by LZTFL1 via CTNNB1 (generic binding protein) and MPPs (metalloprotease) fits with their protections against the fatal comorbidity.

More interestingly, nine of the ten members in this network, along with LRP1 and PARP1 in the TMPRSS2 pathway, had nominal significance for genetic associations indeed with the severity of COVID-19 (Fig. 1 legend for *left panel*), as revealed by the meta-analysis of GWAS [18]. For *APOE*, it was rs429358 that encodes APOE4 (*p*_*meta*_ = 0.0026), but not another nearby (only 138 bp away) SNP rs7412 C/T that differentiates the nonpathogenic APOE2 vs 3 (Cys176Arg) (*p*_*meta*_ = 0.73), that showed an association with severe COVID-19, selectively supporting the underlying risk of APOE4 and the in vitro experimental finding that there is an association between APOE4 and COVID-19 infectivity. The APOE networking had a 17.6-fold enrichment for associations with severe COVID-19 based on *p*_*meta*_-values, comparing to the whole GWAS, pointing to a shared molecular etiology.

It remains unknown how significant this pathway information contributes to ACE2/TMPRSS2/NRP1-related infection itself. Such information however encourages modeling analysis of human peripheral (epithelial and immune) cells that bear the brunt of the coronavirus infection for further clarification of the APOE4 mechanism in COVID-19 development and progression. Even worse for AD, the molecular vulnerability can be furthered by exposure-related behavioral disadvantage in the patients (*right panel* in Fig. 1).

## Conclusion

**Fig. 2:**
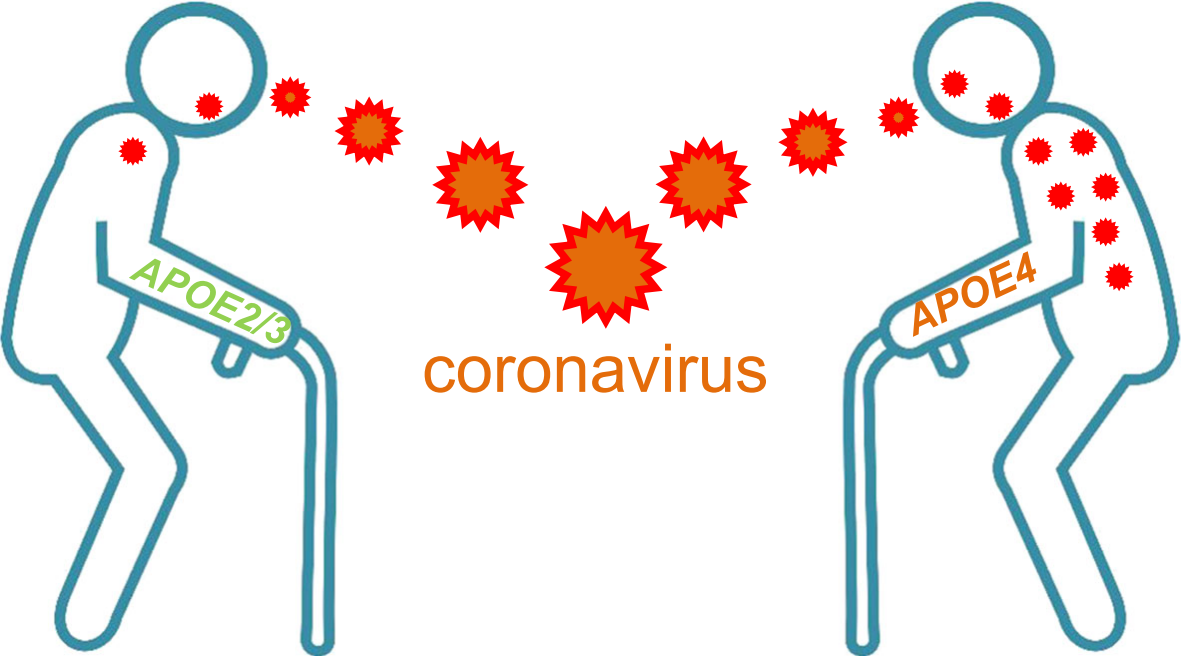
Summary: coronavirus targets AD patients carrying APOE4

Comparing to two other isoforms, APOE4 is genetically associated with reduced APOE levels for increased coronavirus infection and disease progression risks, and consistently with severe COVID-19 as well. As summarized in Fig. 2, the association between APOE4 and coronavirus infectivity supports the hypothesis that APOE4 is an important risk marker for the severity of COVID-19 pathology in patients with AD. If further verified, APOE genotyping may help guide evidence-based healthcare of the comorbid patients.

## Data Availability

The corresponding author has the data available.
